# 25(OH) Vitamin D Levels and Severity of Type 1 Diabetes in Youth and Adults With Recent-Onset Disease

**DOI:** 10.1210/jendso/bvaf061

**Published:** 2025-04-10

**Authors:** David A Baidal, Ana M Alvarez, Nathalia Padilla, Janine Sanchez, Giacomo Lanzoni, Rodolfo Alejandro, Camillo Ricordi

**Affiliations:** Diabetes Research Institute, Miller School of Medicine, University of Miami, Miami, FL 33136, USA; Department of Medicine, Division of Endocrinology, Diabetes and Metabolism, University of Miami, Miami, FL 33136, USA; Diabetes Research Institute, Miller School of Medicine, University of Miami, Miami, FL 33136, USA; Diabetes Research Institute, Miller School of Medicine, University of Miami, Miami, FL 33136, USA; Diabetes Research Institute, Miller School of Medicine, University of Miami, Miami, FL 33136, USA; Department of Pediatrics, University of Miami, Miami, FL 33136, USA; Diabetes Research Institute, Miller School of Medicine, University of Miami, Miami, FL 33136, USA; Department of Biochemistry and Molecular Biology, University of Miami, Miami, FL 33136, USA; Diabetes Research Institute, Miller School of Medicine, University of Miami, Miami, FL 33136, USA; Department of Medicine, Division of Endocrinology, Diabetes and Metabolism, University of Miami, Miami, FL 33136, USA; Diabetes Research Institute, Miller School of Medicine, University of Miami, Miami, FL 33136, USA; DeWitt Daughtry Family Department of Surgery, Division of Cellular Transplantation, University of Miami, Miami, FL 33136, USA

**Keywords:** recent-onset type 1 diabetes, vitamin D, C-peptide, mixed meal tolerance test

## Abstract

**Objective:**

To test 25-hydroxy vitamin D (25(OH)D) levels among subjects with new-onset type 1 diabetes (T1D) and their association with fasting and stimulated C-peptide at study entry in an open-label randomized trial.

**Methods:**

We conducted a post hoc secondary analysis of the POSEIDON trial (a Pilot, Safety and Feasibility Trial of High-Dose Omega-3 fatty acids and High-Dose Cholecalciferol Supplementation in Type 1 Diabetes). Eligibility criteria included age 6 to 65 years, T1D of up to 10 years duration, presence of at least 1 islet autoantibody, and stimulated C-peptide ≥0.066 pmol/mL. A total of 18 subjects with new-onset T1D (defined as ≤180 days duration) with paired 25(OH)D levels and a 4-hour mixed meal tolerance test (MMTT) at screening were included.

**Results:**

25(OH)D levels were directly associated with fasting C-peptide (*r* = 0.589; 95% CI 0.154-0.833; *P* = .01) but no significant associations were found with MMTT stimulated C-peptide. Ten subjects had 25(OH)D levels <30 ng/mL (56%) and fasting C-peptide was significantly lower compared to those with 25(OH)D levels >30 ng/mL (0.22 ± 0.14 vs 0.41 ± 0.09 pmol/mL; *P* < .006).

**Conclusion:**

25(OH)D levels were directly associated with fasting C-peptide in youth and adults with newly diagnosed T1D. Low 25(OH)D levels may be associated with more aggressive autoimmunity in patients at risk for T1D potentially leading to a lower beta-cell mass at T1D clinical onset, but larger studies are required to validate these results.

The POSEIDON study (a Pilot, Safety and Feasibility Trial of High-Dose Omega-3 fatty acids and High-Dose Cholecalciferol Supplementation in Type 1 Diabetes; NCT03406897) was conducted to evaluate whether combination therapy with omega-3 fatty acids and cholecalciferol could be of assistance in halting progression of immune-mediated pancreatic islet loss and preserve beta-cell function in subjects with new-onset and established type 1 diabetes (T1D) of up to 10 years duration [1]. The study has been completed, and results are currently being analyzed. Herein, we report on a post hoc secondary analysis evaluating 25-hydroxyvitamin D (25(OH)D) levels among subjects with new-onset T1D and their association with fasting C-peptide and mixed meal tolerance test (MMTT)-stimulated C-peptide measures at study entry.

## Materials and Methods

### Study Design and Patients

The study was approved by the University of Miami Institutional Review Board and was registered with ClinicalTrials.gov (NCT03406897). The full protocol has been previously published [1]. All subjects (or parents) provided written informed consent and subjects <18 years of age signed a study assent. Briefly, eligibility criteria included an age between 6 and 65 years, a diagnosis of T1D with duration of up to 10 years before randomization, the presence of at least 1 islet autoantibody, and a stimulated C-peptide of at least 0.066 pmol/mL during a 4-hour MMTT. Key exclusion criteria included concomitant therapy with immunosuppressive drugs, immunomodulators, or cytotoxic agents, history of gastroparesis or other severe gastrointestinal disease, coagulation or bleeding disorders, and contraindications to omega-3 fatty acids and/or vitamin D supplements. New-onset T1D was defined as T1D of ≤180 days duration, whereas established disease was defined as T1D of >180 days duration.

### C-Peptide and Vitamin D Assays

C-peptide levels were measured from serum using an electrochemiluminescence immunoassay (Elecsys C-peptide, Cobas, Roche Diagnostics, Indianapolis, IN; RRID:AB_2909476). 25(OH)D levels were measured using an electrochemiluminescence binding assay (Elecsys Vitamin D total III, Cobas, Roche Diagnostics, Indianapolis, IN; RRID:AB_2909604).

### Mixed Meal Tolerance Test

Subjects were required to fast overnight. The test started before 10 Am and fasting glucose was required to be 70 to 200 mg/dL. Long-acting insulin or basal insulin via insulin pump were permitted but no rapid-acting insulin boluses or corrections were allowed within 2 hours of the start of the test. Boost® High Protein Nutritional Drink (Nestlé, Health Science S.A.) was used at a dose of 6 mg/kg to a maximum of 360 mL and subjects were required to ingest the liquid meal over 5 minutes. Samples for glucose and C-peptide were obtained at −10, 0, 15, 30, 60, 90, 120, 150, 180, 210, and 240 minutes after ingestion. Vitamin D levels were measured from a fasting sample collected prior to starting the MMTT.

### Statistical Analyses

Data are presented as means and SD. Spearman correlation was used to assess the relationship between continuous variables. Mann-Whitney U-test was used to evaluate differences in medians as data was not normally distributed. The area under the curve (AUC) was computed using the trapezoidal rule calculated by the sum of the timed C-peptide values during the MMTT test, then divided by 240 (minutes). Analyses were performed with GraphPad Prism version 6.07.

## Results

### Patient Characteristics and Baseline Values

A total of 19 subjects with new-onset T1D underwent screening and had paired 25(OH)D levels and 4-hour MMTT data. One subject, a 10-year-old boy, was excluded from these analyses as he had been on high-dose vitamin D supplements prior to study enrollment and his 25(OH)D level at screening was markedly elevated at 127 ng/mL. Of the 18 subjects evaluated, 13 were male (72%), 13 were <18 years of age (72%), 10 had 25(OH)D levels <30 ng/mL (56%), and 2 had 25(OH)D levels <20 ng/mL (11%). Diabetes duration was 86 ± 49 days, 25(OH)D level 31.52 ± 10.24 ng/mL, fasting C-peptide 0.31 ± 0.15 pmol/mL, and daily insulin dose 0.35 ± 0.22 units/kg/day (n = 18). Additional patient characteristics by age group are described in Table 1. The youth cohort comprised 13 subjects aged 6 to 16 years, and 7 (54%) were classified as pubertal based on Tanner staging. Daily insulin dose was significantly higher in youth compared to adults (0.42 ± 0.20 vs 0.17 ± 0.19 units/kg/day; *P* = .032). No other significant differences were noted between youth and adults at baseline.

**Table 1.** Baseline characteristics of youth and adults with new-onset type 1 diabetes at screening

	All	Youth	Adults
n	18	13	5
Male, n (%)	13 (72)	10 (77)	3 (60)
Age, years	14.6 ± 9.1	10 ± 3	26 ± 11
Weight, kg	47.5 ± 20.8	38.8 ± 16.3	70.4 ± 11.5
Diabetes duration, days	86 ± 49	81 ± 43	99 ± 64
HbA1c, %	7.3 ± 1.7	7.1 ± 1.6	7.8 ± 2.0
Total daily insulin dose, units/kg/day	0.35 ± 0.22	0.42 ± 0.20	0.17 ± 0.19
25(OH)D, ng/mL	31.5 ± 10.2	33.8 ± 10.4	25.5 ± 7.6
Fasting C-peptide, pmol/mL	0.31 ± 0.15	0.31 ± 0.14	0.30 ± 0.20
MMTT 90 minutes C-peptide, pmol/mL	0.76 ± 0.34	0.76 ± 0.28	0.82 ± 0.50
MMTT 4-hour AUC C-peptide, pmol/mL	0.68 ± 0.30	0.64 ± 0.22	0.78 ± 0.47

Data are presented as means and SD.

Abbreviations: 25(OH)D, 25-hydroxy vitamin D; AUC, area under the curve; HbA1c, glycated hemoglobin; MMTT, mixed meal tolerance test.

### 25(OH)D Levels and Fasting C-Peptide

We evaluated differences in fasting C-peptide based on vitamin D sufficiency status.

**Figure 1.**
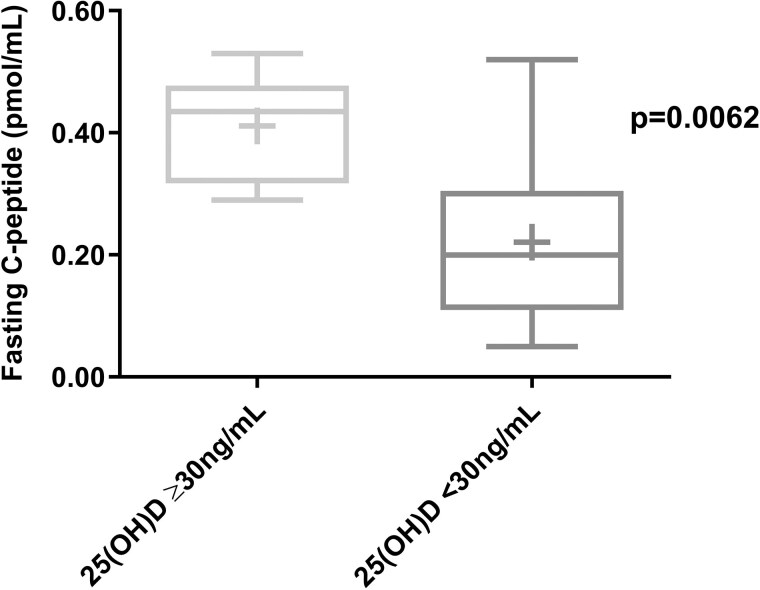
Fasting C-peptide in subjects with new-onset type 1 diabetes according to 25(OH)D level. The + sign denotes the mean value.

In subjects with 25(OH)D levels ≥30 ng/mL (44%), fasting C-peptide was 0.41 ± 0.09 pmol/mL. On the other hand, subjects with 25(OH)D levels <30 ng/mL (56%) had significantly lower fasting C-peptide levels, 0.22 ± 0.14 pmol/mL (*P* < .006) (Fig. 1).

### Correlation Between 25(OH)D Levels and C-Peptide Measures

**Figure 2.**
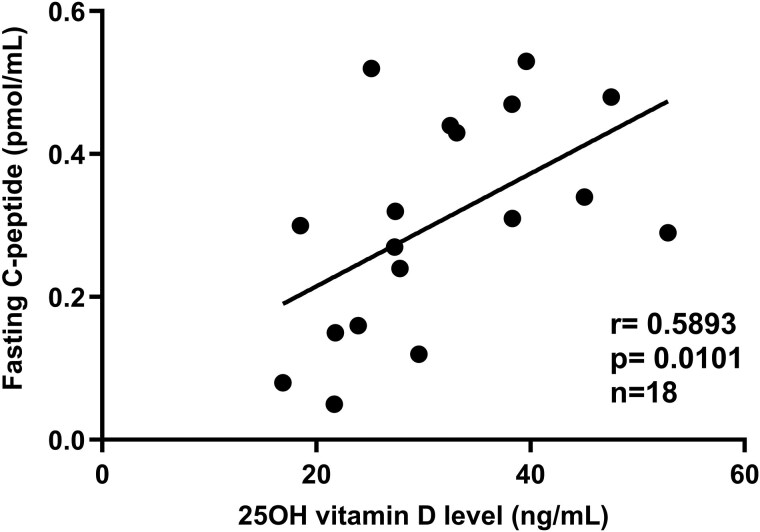
Correlation between 25(OH)D levels and fasting C-peptide in subjects with new-onset type 1 diabetes (<6 months duration) at screening.

25(OH)D levels were directly associated with fasting C-peptide (*r* = 0.589; 95% CI 0.154 to 0.833; *P* = .01) (Fig. 2). By contrast, no significant association was noted between 25(OH)D levels and stimulated C-peptide measures, including the MMTT 90-minute C-peptide (*r* = 0.246; 95% CI −0.269 to 0.6486; *P* = .32) and the 4-hour MMTT AUC C-peptide (*r* = 0.209, 95% CI −0.299 to 0.625; *P* = .41).

## Discussion

Although the conduction of the POSEIDON clinical trial was negatively impacted by the COVID-19 pandemic, which led to delayed and missed study visits, results of testing at screening provide useful insights on the association between 25(OH)D levels and severity of T1D at onset, as reflected by C-peptide levels.

In our study, we found a significant correlation between 25(OH)D levels at T1D onset and fasting C-peptide, with most participants (56%) presenting with vitamin D deficiency (25(OH)D < 30 ng/mL). In addition, fasting C-peptide strongly correlated with MMTT C-peptide stimulated measures. Notably, fasting C-peptide levels in vitamin D–sufficient subjects were 0.41 ± 0.09 pmol/mL (mean ± SD), close to levels reported in patients diagnosed by surveillance (ie, undergoing serial oral glucose tolerance tests prior to diagnosis) who have been reported to present with less severe T1D at diagnosis [2-5]. By contrast, fasting C-peptide in those with 25(OH)D levels <30 ng/mL was 0.22 ± 0.14 pmol/mL (mean ± SD), almost half the value of those with 25(OH)D levels ≥30 ng/mL and similar to previously reported data for patients not enrolled in a surveillance program and diagnosed with T1D [6, 7].

Low levels of vitamin D have been identified at onset of T1D in children and young adults [8-11]. In the TEDDY study, higher childhood 25(OH)D was associated with lower islet autoimmunity risk [12]. Clinical studies in subjects with new-onset T1D have shown that treatment with cholecalciferol (vitamin D3) results in both an increase in the percentage and the suppressive capacity of regulatory T-cells [13, 14]. Daily treatment with 4000 IU of vitamin D3 significantly reduced CD4+ T-cell activation compared to treatment with 400 units of vitamin D3 in adults with vitamin D deficiency [15]. Gabbay et al evaluated the administration of 2000 IU of cholecalciferol daily on residual beta-cell function in subjects with new-onset T1D and showed a higher stimulated C-peptide at 18 months compared to placebo [13]. More recently, Nwosu et al conducted a randomized controlled trial in in youth (aged 10-21 years) with newly diagnosed T1D testing ergocalciferol 50 000 IU given weekly by mouth for 2 months followed by 50 000 IU every other week for 10 months [16]. Although no significant differences were found in stimulated C-peptide at 12 months, a post hoc secondary analysis showed that treatment with ergocalciferol significantly slowed the decline in percentage AUC C-peptide from baseline compared with placebo (*P* = .03) [17].

In addition, several studies have explored the relationship between vitamin D levels and islet autoimmunity highlighting the significant role of vitamin D in modulating the immune system and its potential protective effects against autoimmune diseases [18-20]. Of note, the VITAL trial demonstrated that daily supplementation of vitamin D (cholecalciferol; 2000 IU) over a 5-year period reduced the risk of developing autoimmune diseases by 22% in older adults [21]. Further, a preplanned analysis excluding the first 2 years of follow-up to test the latency of treatment effects showed a significantly lower incidence of confirmed autoimmune disease in the vitamin D group compared to placebo (hazard ratio 0.61; 95% CI 0.43 to 0.86; *P* = .005).

Given the small sample size, our data is hypothesis-generating and not conclusive, and it suggests that low vitamin D levels may be associated with more aggressive autoimmunity in patients at risk for T1D potentially leading to a lower beta-cell mass at T1D clinical onset (stage 3) as evidenced by lower fasting C-peptide levels. The assessment of vitamin D status and correction of vitamin D deficiency in subjects at risk for T1D (stage 1, stage 2) may be of importance in the progression to stage 3 T1D.

The main limitations of our analyses are the small sample size, limiting their generalizability, and the absence of longitudinal data for 25(OH)D and fasting C-peptide levels prior to disease onset. In addition, gender, age, pubertal status, and glycemic control may have had an impact in the relationship between 25(OH)D and C-peptide levels. In view of the small sample size, we were unable to adjust for these potential confounders. However, age and glycated hemoglobin (HbA1c) were not significantly correlated with 25(OH)D and fasting C-peptide (data not shown). On the other hand, the main strength of our data is the availability of paired 25(OH)D levels with fasting and stimulated C-peptide measures in youth and adults with new-onset T1D.

## Conclusions

In this pilot study, 25(OH)D levels were positively associated with fasting C-peptide at T1D onset and most of the participants were vitamin D–deficient. Low 25(OH)D levels may be associated with more aggressive autoimmunity in patients at risk for T1D, potentially leading to a lower beta-cell mass at T1D clinical onset. Prospective trials are required to assess whether correction of vitamin D deficiency in early T1D stages may delay progression to stage 3 and/or allow for a slower rate of C-peptide decline following disease onset.

## Data Availability

Datasets generated during and/or analyzed during the current study are not publicly available but are available from the corresponding author on reasonable request.

## Clinical Trial Information

Clinical trial registration number: NCT03406897.

## Abbreviations

25(OH)D: 25-hydroxyvitamin D
AUC: area under the curve
MMTT: mixed meal tolerance test
T1D: type 1 diabetes
